# Chemokine Signaling during Midline Epithelial Seam Disintegration Facilitates Palatal Fusion

**DOI:** 10.3389/fcell.2017.00094

**Published:** 2017-10-30

**Authors:** Christiaan M. Suttorp, Niels A. Cremers, René van Rheden, Raymond F. Regan, Pia Helmich, Sven van Kempen, Anne M. Kuijpers-Jagtman, Frank A.D.T.G. Wagener

**Affiliations:** ^1^Department of Orthodontics and Craniofacial Biology, Radboud University Medical Centre, Nijmegen, Netherlands; ^2^Radboud Institute for Molecular Life Sciences, Radboud University Medical Centre, Nijmegen, Netherlands; ^3^Department of Rheumatology, Radboud University Medical Centre, Nijmegen, Netherlands; ^4^Department of Emergency Medicine, Thomas Jefferson University, Philadelphia, PA, United States

**Keywords:** embryology, cleft palate, chemokine, macrophage, heme oxygenase, apoptosis

## Abstract

Disintegration of the midline epithelial seam (MES) is crucial for palatal fusion, and failure results in cleft palate. Palatal fusion and wound repair share many common signaling pathways related to epithelial-mesenchymal cross-talk. We postulate that chemokine CXCL11, its receptor CXCR3, and the cytoprotective enzyme heme oxygenase (HO), which are crucial during wound repair, also play a decisive role in MES disintegration. Fetal growth restriction and craniofacial abnormalities were present in HO-2 knockout (KO) mice without effects on palatal fusion. CXCL11 and CXCR3 were highly expressed in the disintegrating MES in both wild-type and HO-2 KO animals. Multiple apoptotic DNA fragments were present within the disintegrating MES and phagocytized by recruited CXCR3-positive wt and HO-2 KO macrophages. Macrophages located near the MES were HO-1-positive, and more HO-1-positive cells were present in HO-2 KO mice compared to wild-type. This study of embryonic and palatal development provided evidence that supports the hypothesis that the MES itself plays a prominent role in palatal fusion by orchestrating epithelial apoptosis and macrophage recruitment via CXCL11-CXCR3 signaling.

## Introduction

Formation of the secondary palate requires adhesion by the midline epithelial edge (MEE) of both palatal shelves, formation of the transient midline epithelial seam (MES), disintegration of the MES, and fusion of the palatal shelves (Ackermans et al., 2011). Only after disintegration of the MES the mesenchyme of the palatal shelves can fuse to form the secondary palate. Failure of epithelial adhesion between both palatal shelves (Dudas et al., 2006) or a lack of MES disintegration (Gritli-Linde, 2007; Iseki, 2011) will result in cleft palate with (CLP) or without cleft lip (CPO). Multiple mechanisms have been proposed to explain the disappearance of the MES. The main hypotheses underlying MES disintegration involve epithelial cell migration to the oral or nasal epithelium (Jin and Ding, 2006), epithelial-to-mesenchymal transformation (EMT) (Nawshad, 2008), epithelial cell apoptosis (Vaziri Sani et al., 2005; Xu et al., 2006; Vukojevic et al., 2012; Lan et al., 2015), or a combination of these events (Iseki, 2011).

CLP is the most common congenital facial malformation in humans and occurs in approximately 1/700 live births (Brown and Sandy, 2007). However, CPO is the rarest form of oral clefting, with an incidence ranging from 1.3 to 25.3/10,000 live births (Burg et al., 2016). Although the exact biological mechanisms underlying orofacial clefting are not completely understood (Mossey et al., 2009), a combination of genetic and environmental factors is thought to play a role. Approximately 50% of children born with CPO have a genetic syndrome (Watkins et al., 2014), compared to 30% with CLP (Drew, 2014). Notably, maternal smoking, diabetes, and infections have been shown to strongly increase the risk for babies with orofacial clefts (Mossey et al., 2009; Brocardo et al., 2011), suggesting that control of oxidative and inflammatory stress is important.

Accumulating data suggest that the heme-degrading antioxidative enzyme heme oxygenase (HO) is a key regulator during embryological development (Zenclussen et al., 2003, 2011, 2014). HO facilitates placentation, fetal growth, and -development by restricting excessive free heme levels. Heme promotes oxidative and inflammatory stress (Wagener et al., 2001, 2010) and may lead to intrauterine fetal growth restriction and fetal loss (Zenclussen et al., 2011). HO protects against this inflammatory stress by degrading heme and generating free iron/ferritin, carbon monoxide (CO), and biliverdin/bilirubin (Wagener et al., 2003). These HO effector molecules regulate vasodilation and anti-apoptotic signaling, inhibit platelet aggregation, reduce leukocyte adhesion, and reduce pro-inflammatory cytokines (Wagener et al., 2003; Grochot-Przeczek et al., 2012). Two functional isoforms of HO have been described, HO-1 and HO-2. HO-1 has low basal levels but is strongly inducible, whereas HO-2 is largely constitutively expressed. HO-2 is highly expressed in the brain, testes, and blood vessels (Ewing and Maines, 2006). Interestingly, the cytoprotective HO-1 and HO-2 enzymes are both strongly expressed in the placenta during embryonic development and in neural crest cells that form the craniofacial tissues in mice and humans (Zenclussen et al., 2003; Shi et al., 2008). Elevated inflammatory, oxidative, and angiogenic factors have been demonstrated in the endothelial cells of HO-2 knockout (KO) mice (Bellner et al., 2009). During pregnancy, down-regulation of both HO-1 (Sollwedel et al., 2005) and HO-2 (Zenclussen et al., 2003) in the placenta is associated with pregnancy failure. Spontaneous abortion, pre-eclampsia, or fetal growth retardation are associated with lower HO-2 protein levels compared to healthy pregnant controls (Zenclussen et al., 2003).

Palatal fusion and wound repair are regenerative processes that share common signaling pathways and gene regulatory networks (Biggs et al., 2015). Epithelial-mesenchymal cross-talk is essential in both processes. Decreased signaling between the versatile CXCL11 and its receptor CXCR3 on epithelial cells leads to delayed re-epithelialization and impaired epidermis maturation during wound repair (Satish et al., 2005). Moreover, CXCR3 KO mice present excessive scar formation following injury (Yates et al., 2010). Macrophages are important in regenerative and embryonic developmental processes (Epelman et al., 2014, 2015; Roszer, 2015; Mass et al., 2016), and blocking the CXCL11-CXCR3 axis suppresses macrophage infiltration (Kakuta et al., 2012; Torraca et al., 2015). Recently, slower wound closure and delayed CXCL11 expression in wounds was observed in HO-2 KO mice (Lundvig et al., 2014). Interestingly, HO-2 deficiency was shown to result in impaired macrophage function (Bellner et al., 2015). Although CXCL11-CXCR3 signaling regulates diverse cellular functions, including influx of immune cells during inflammation (Kaplan, 2001; Van Raemdonck et al., 2015) and wound repair (Satish et al., 2005; Balaji et al., 2015), little is known about its role during palatal fusion.

We postulate that palatal fusion is hampered in HO-2 KO mice by disruption of epithelial cell and macrophage cross-talk in the MES due to hampered CXCL11-CXCR3 signaling. In the present study, MES disintegration is investigated in relation to chemokine signaling and the effects of HO-2 deficiency on embryonic development and palatal fusion.

## Materials and methods

### Animals used for the study

To obtain fetuses for this study, 8-week-old female wild type (wt) (*n* = 7) and HO-2 KO (*n* = 8) mice were mated with respectively wt and HO-2 KO male mice. Homozygote HO-2 KO mice generated by targeted disruption of the HO-2 gene (Poss et al., 1995; Bellner et al., 2009), and wt mice, both of a mixed 129Sv × C57BL/6 background, were bred and maintained in our animal facility. The animals were housed under normal laboratory conditions with 12 h light/dark cycle and *ad libitum* access to water and powdered rodent chow (Sniff, Soest, The Netherlands) and were allowed to acclimatize for at least 1 week before the start of the experiment. Ethical permission for the study was obtained according to the guidelines of the Board for Animal Experiments of the Radboud University Nijmegen (RU-DEC 2012-166).

### Hormone administration before mating

Preliminary experiments (RU-DEC 2009-160) demonstrated that young animals (8–10 weeks old), that were mated for the first time, often did not carry fetuses. This was demonstrated for both wt mice and HO-2 KO mice. The chance of pregnancy was therefore enhanced using the hormones Folligonan (Genadotropin serum, Intervet Nederland B.V., Boxmeer, The Netherlands) and Pregnyl (Human chorionic gonadotropin, N.V. Organon, Oss, The Netherlands). Because there is a lag time period of approximately 13 days between hormone application and palatal formation, we expected minor influence on the experimental outcome. At day −3 at 16.00 h Folligonan (6E in 30 μl) and at day −1 at 16.00 h Pregnyl (6E in 30 μl) was administered by intraperitoneal injection.

### Plugging day and obtaining wt and HO-2 KO fetuses

The presence of a vaginal copulation plug, indicating that mating has occurred, was taken as day 0 of pregnancy (embryonic day 0; E0) (Behringer et al., 2016). 1 wt mouse and 2 HO-2 KO mice demonstrated no plugged status. These animals were mated again 4 weeks later, and all demonstrated then a plugged status.

Since the palatal shelves fuse between embryonic day E14.5 and E15.5 in wt mice (Dudas et al., 2007) we presumed that fetuses of E15 were suitable for our study. At embryonic day E15, 7 wt and 7 HO-2 KO animals were killed by CO_2_/O_2_ inhalation for 10 min. Only 3 out of 7 plugged wt mice, and 4 out of 7 plugged HO-2 KO mice carried fetuses. In total, 16 wt fetuses of E15 and 11 HO-2 KO fetuses of E15 were obtained.

In order to monitor the growth restriction found in HO-2 KO fetuses in more detail, we also analyzed the body size of E16 HO-2 KO fetuses. Therefore, 1 pregnant HO-2 KO mouse was killed at embryonic day 16, resulting in 12 HO-2 KO fetuses of E16.

### Implantation rate

**Figure 1:**
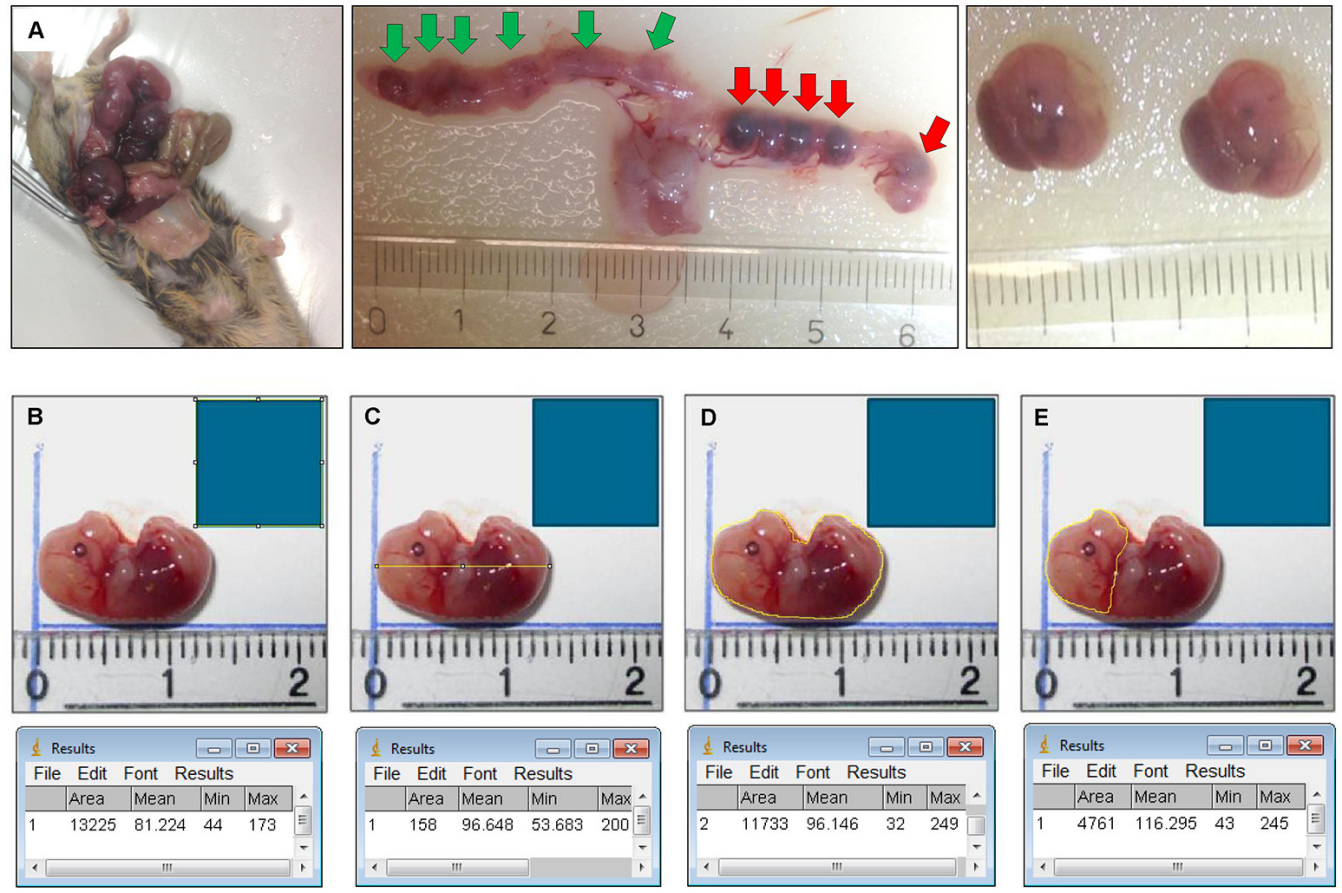
**(A)** Isolation of the fetuses and measurement of body length, body surface, head surface. Plugged mouse (e.g., HO-2 KO at E15) was sacrificed by CO_2_/O_2_ inhalation for 10 min, the uterus and organs were removed. Fetuses were isolated from the uterus. Location of the 6 fetuses in the uterus before they were removed (green arrows). Location of the 5 non-viable/hemorrhagic embryonic implantations (red arrows). **(B)** A square scale bar was drawn at the ruler in each photograph of 10 × 10 mm (1 cm^2^) and the total number of pixels within the square was determined (e.g. 13,225 pixels). **(C)** A line in the length of the body of the fetus was drawn and the number of pixels was recorded (e.g. 158 pixels). The length was calculated (e.g. 158/√13,225 = 13,7 mm). **(D)** The outline of the total body surface of the fetus was drawn and the number of pixels was recorded (e.g. 11,733 pixels). The total body surface was calculated (e.g. 11,733/13,225 = 0.89 cm^2^). **(E)** The outline of the head surface was drawn and the number of pixels was recorded (e.g. 4,761 pixels). The head surface was calculated (e.g. 4761/13,225 = 0.36 cm^2^).

The uterus and fetuses were photographed (Figure 1). For the fetus carrying mice the mean implantation rate was analyzed by calculating the percentage of fetuses to the total number of embryonic implantations (fetuses+non-viable or hemorrhagic embryonic implantations).

### The wt and HO-2 KO fetuses compared for weight, length, and body surface

**Figure 2:**
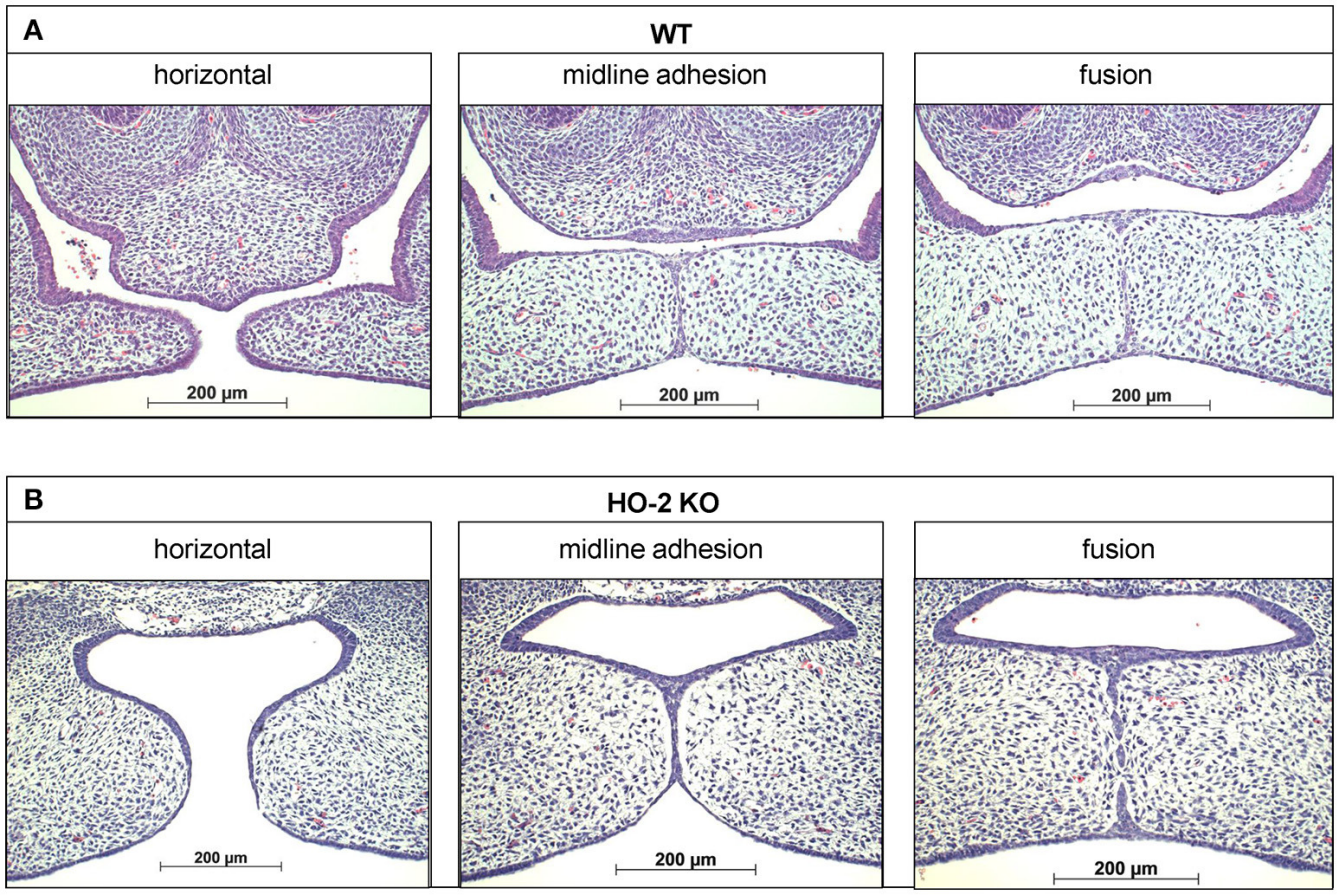
Palatal fusion observed in both wt and HO-2 KO fetuses at E15. HE stainings demonstrated horizontal orientation of the palatal shelves, midline adhesion and fusion within the same fetus. **(A)** Wt fetus at E15 (magnification: x100). Palatal shelves in a later stage of the palatal fusion increased in size. The MES changed from a multi-cell-layer into a continuous one-single-cell-layer, to a disintegrating MES, during which islands of epithelium in the midline were observed. **(B)** HO-2 KO fetus at E15 (magnification: x100). Palatal shelves in a later stage of the palatal fusion increased to some extent in size. Several islands of epithelium in the midline were observed.

The weight and size of wt fetuses (E15) and HO-2 KO fetuses (E15/E16) was measured. Severely malformed fetuses (*n* = 2) were excluded for weight and size analysis. The body length, body surface and head surface of the fetuses were measured from photographs using ImageJ (1.48 v) software (National institutes of health, Bethesda, MD, USA) and used for statistical analysis, for details see Figure 2.

### Assessment of mRNA expression of HO-2, CXCL11, CXCR3, and HO-1 in fetus head samples by quantitative real-time PCR

To screen for differences in gene expression between HO-2 KO and wt fetuses, cDNAs were synthesized from samples from heads of wt (E15; *n* = 5) and HO-2 KO (E15; *n* = 4) fetuses. Fetuses were decapitated and total RNA was extracted using Trizol (Invitrogen, Carlsbad, CA, USA) and a RNeasy Mini kit (Qiagen, Hilden, Germany), and cDNA was produced using the iScript cDNA synthesis kit (Bio-Rad, Hercules, CA, USA). cDNAs were analyzed for gene expression of HO-1, HO-2, CXCL11, and CXCR3, using custom-designed primers (Table 1) and iQ SYBR Green Supermix (Invitrogen, Carlsbad, CA, USA) in a CFX96 real-time PCR system (Bio-Rad, Hercules, CA, USA). Relative gene expression values were evaluated with the 2^∧(−ΔΔCt)^ method using GAPDH as housekeeping gene (Livak and Schmittgen, 2001).

**Table 1:** Custom-designed mouse primers used for assessment of mRNA expression of GAPDH, HO-1, HO-2, CXCL11, and CXCR3 in fetus head samples by quantitative real-time PCR.

**Marker**	**Gene Name**	**Forward primer (5′-3′)**	**Reverse primer (5′-3′)**
Reference gene	*Gapdh*	GGCAAATTCAACGGCACA	GTTAGTGGGGTCTCGCTCCTG
Cytoprotection	*Hmox1*	CAACATTGAGCTGTTTGAGG	TGGTCTTTGTGTTCCTCTGTC
Cytoprotection	*Hmox2*	AAGGAAGGGACCAAGGAAG	AGTGGTGGCCAGCTTAAATAG
Chemokine	*Cxcl11*	CACGCTGCTCAAGGCTTCCTTATG	TGTCGCAGCCGTTACTCGGGT
Chemokine receptor	*Cxcr3*	CAGCCTGAACTTTGACAGAACCT	GCAGCCCCAGCAAGAAGA

### Haematoxylin-eosin staining of transversal sections through the secondary palate

Mouse tissue samples were fixed for 24 h in 4% paraformaldehyde and further processed for routine paraffin embedding. Paraffin sections were deparaffinized using Xylol, rehydrated using an alcohol range (100–70%), and used for immunohistochemistry and FragEL™ analysis. Serial transversal sections through the secondary palate region of 5 μm thickness mounted on Superfrost Plus slides (Menzel-Gläser, Braunschweig, Germany) were routinely stained with Haematoxylin and Eosin (HE) for general tissue survey. The exact location of the fusing palatal shelves was determined per individual fetus. The HE stainings were subdivided into the four stages of palatogenesis based on the anatomy of the palatal shelves: elevation, horizontal, midline adhesion and fusion, according to Dudas et al. (2007) and screened for anatomical abnormalities. These series were used as reference to obtain transversal sections containing palatal shelves in midline adhesion and fusion for immunohistochemical staining (Figure 2).

### Immunohistochemistry

Selected paraffin embedded sections were deparaffinized using Xylol and rehydrated using an alcohol range (100–70%). Endogenous peroxidase activity was quenched with 3% H_2_O_2_ in methanol for 20 min, and immunohistochemical stainings for HO-1, CXCL11, CXCR3, and macrophage marker F4/80 were performed as previously described (Tan et al., 2009). In brief, tissue sections were incubated for 60 min with a biotin-labeled secondary antibody (Table 2). Next, the sections washed with PBSG (phosphate-buffered saline with glycine) and treated with avidin-biotin peroxidase complex (ABC) for 45 min in the dark. After extensive washing with PBSG, diaminobenzidine-peroxidase (DAB) staining was performed for 10 min for the HO-1, CXCL11, and CXCR3 stainings.

**Table 2:** Antibodies and antigen retrievals used for immunohistochemical stainings for HO-1, CXCL11, CXCR3 and F4/80.

**Antibody**	**Specificity**	**Dilution**	**Antigen retrieval**	**Source**
SPA-895	HO-1	1:600	Combi: Citrate buffer 70°C for 10 min	Stressgen
			Trypsin digestion in PBS 0.015% 37°C for 5 min	
Sc-34785	CXCL11	1:200	Citrate buffer 70°C for 2 h	Santa Cruz Biotechnology, Santa Cruz, CA, USA
NB100-56404	CXCR3	1:50	Citrate buffer 70°C for 2 h	Novus Biologicals, Littleton, USA
BM8	F4/80	1:400	Combi: Citrate buffer 70°C for 10 min	eBioscience
			Trypsin digestion in PBS 0.015% for 5 min	

### Analysis of apoptosis and recruited F4/80 positive macrophages in the palate

For studying apoptosis in the MES, transversal sections containing palatal shelves in midline contact and fusion stage of wt and HO-2 KO fetuses were selected. During apoptosis, cellular endonucleases cleave nuclear DNA between nucleosomes, producing specific DNA fragments with free 3′-OH groups at the end. These 3′ OH group can be labeled using Fragment End Labeling (FragEL™, Calbiochem, San Diego, CA, USA) allowing detection of apoptotic DNA fragments at the individual cell level as previously described (Siemieniuch, 2008). The procedure was performed according to the protocol of the manufacturer (Calbiochem, San Diego, CA, USA). In brief, rehydrated paraffin sections were subjected to proteinase K digestion (0.5 μg/ml) for 10 min. Endogenous peroxidase activity was quenched with 3% H_2_O_2_ in methanol for 20 min. TdT (Terminal deoxynucleotidyl Transferase) added biotin labeled deoxynucleotides to the end of these DNA fragments. After addition of ABC, DAB was added and incubated at room temperature for 10 min. For the F4/80 staining + AP (alkaline phosphatase) + NBT (nitro-blue tetrazolium) + BCIP (5-bromo-4-chloro-3′-indolyphosphate) was used. For used antibodies and antigen retrievals, see Tables 2, 3. Photographs were taken using a Carl Zeiss Imager Z.1 system (Carl Zeiss Microimaging Gmbh, Jena, Germany) with AxioVision (4.8 v) software (Zeiss, Göttingen, Germany).

**Table 3:** Secondary antibodies used for HO-1, CXCL11, CXCR3, apoptotic DNA fragments immunohistochemical staining, and F4/80 with apoptotic DNA fragments/CXCR3/HO-1 double stainings.

**Secondary antibody**	**Specificity**	**Dilution**	**Color**	**Source**
A11008	Goat anti-rabbit AlexaFluor-488	1:500	Green	Invitrogen Thermofisher scientific
145-712-065-153 in combination with S-11226	Donkey anti-rat Biotin	1:500	-	Jackson Immunoresearch Europe LTD Invitrogen Thermofisher scientific
	Streptavidin AlexaFluor-568	1:500	Red	
715-065-151	Donkey anti-rat	1:500	Brown	Jackson Immunoresearch Europe LTD
	Biotin+ABC+DAB			
	Donkey anti-rat	1:500	Blue	Calbiochem, San Diego, CA, USA
	Biotin+ABC+AP+nbt/bcip			

*Antigen retrieval used for double stainings: Combi, Citrate buffer 70°C for 10 min + Trypsin digestion in PBS 0.075% 37°C for 5 min*.

### Quantification of CXCL11, CXCR3 and HO-1 immunoreactivity within the epithelium of the palatal shelves

Transversal sections through the secondary palate of wt and HO-2 KO fetuses were screened. For quantification sections were selected following the inclusion criteria: transversal sections containing palatal shelves in midline adhesion and fusion with at least the presence of a part of the MES.

**Figure 3:**
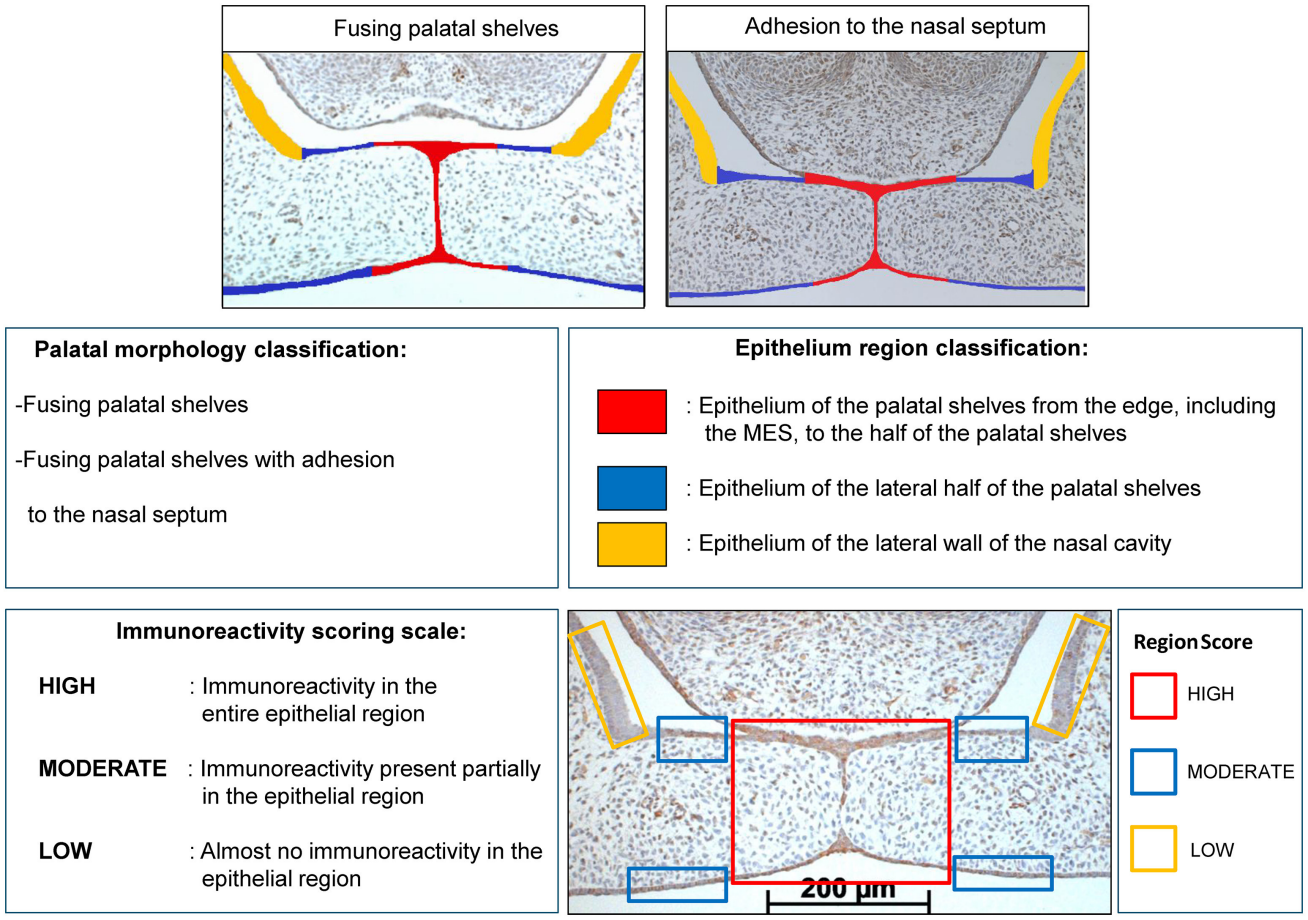
Palatal morphology classification: The CXCL11, CXCR3 and HO-1 immunostained sections were categorized in two stages according to their morphological characteristics: fusing palatal shelves, and fusing palatal shelves with adhesion to the nasal septum. Epithelium region classification: For each section epithelial layers were subdivided in 3 regions of interest according to morphological characteristics: epithelium of the palatal shelves from the edge, including the MES, to half of the width of the shelves (in RED), epithelium of the lateral half of the palatal shelves (in BLUE), epithelium of the lateral wall of the nasal cavity, this region is positioned outside the palatal shelves and served as a control region (in YELLOW). Immunoreactivity scoring scale: Semi-quantitative scoring of CXCL11, CXCR3 and HO-1 immunoreactivity in epithelium of the palatal shelves. Each epithelial region was semi-quantitatively scored according to the following scale: HIGH, Immunoreactivity present in the entire epithelial region; MODERATE, Immunoreactivity present only partially in the epithelial region; LOW, Almost no immunoreactivity present in the epithelial region. Right lower panel: Immunoreactivity scored for the 3 epithelial locations in a CXCL11 immunostained section (e.g. wt fetus, E15, section with adhesion of the palatal shelves and adhesion to the nasal septum). RED region was scored as HIGH, BLUE region was scored as MODERATE, YELLOW region was scored as LOW for CXCL11 immunoreactivity.

The immunostained sections were first categorized into two categories based on their palatal morphology (palatal morphology classification): fusing palatal shelves, and fusing palatal shelves with adhesion to the nasal septum, see Figure 3.

Within each section the epithelium of the palatal shelves was then subdivided into three separate regions (Figure 3) according to morphological characteristics (Epithelium region classification): epithelium of the palatal shelves from the edge, including the MES, to the half of the width of the shelves (in RED), epithelium of the lateral half of the palatal shelves (in BLUE), epithelium of the lateral wall of the nasal cavity, this region is positioned outside the palatal shelves and served as a control region (in YELLOW), see Figure 3.

CXCL11, CXCR3, and HO-1 immunoreactivity was evaluated by two observers, by blindly scoring, independently of each other. The epithelial regions per single section were semi-quantitatively scored according to the immunoreactivity scoring scale in three categories (HIGH, MODERATE, and LOW). For each individual fetus the modus of the scoring per epithelial region was used for further statistical analysis. For details see Figure 3. To determine the inter-examiner reliability, 10 sections were measured by the two observers and acceptable coefficient of determination (*R*^2^) scores >0.80 were obtained for immunoreactivity scoring.

### Quantification of CXCL11, CXCR3, and HO-1 positive cells in the mesenchyme of the palatal shelves

**Figure 4:**
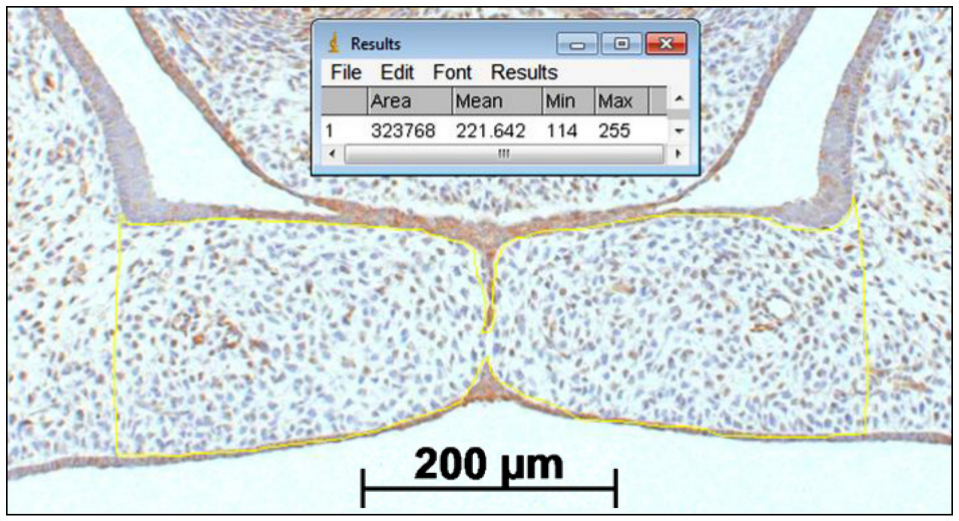
Palatal shelf surface measurement for determining the number of CXCL11, CXCR3 and HO-1 positive immunostained cells cells/mm^2^ within the mesenchyme of the palatal shelves. A square scale bar was drawn in the microscopic picture (magnification: x100) of the section of 1,000 × 1,000 μm (1 mm^2^) and the total number of pixels within the square was determined (e.g. 1 mm^2^ = 3,442,880 pixels). The contour of the mesenchyme of the shelves was drawn (yellow line). The number of pixels for this area was determined by the ImageJ (1.48 v) software (323,768 pixels). The number of positive immunostained cells within this mesenchymal area of the palatal shelves were counted by direct observation using the Zeiss microscope (e.g. 52 CXCL11 positive cells). The number of cells/mm^2^ was calculated (3,442,880/323,768 × 52 = 553 cells/mm^2^).

The immunostained sections, wt and HO-2 KO, were first categorized based on their morphology (Palatal morphology classification, Figure 3). Since a significant variance in the size of the palatal shelves was present, expression was adjusted to surface area. The individual surface of each pair of shelves was measured using ImageJ (1.48 v) software (Zeiss, Göttingen, Germany). Then, the number of CXCL11, CXCR3 and HO-1 positive cells within the outline of the mesenchyme of the palatal shelves was counted. For details see Figure 4. For each section the number of positive mesenchymal cells/mm^2^ was calculated. Cell counting was performed twice, by two blinded observers independently of each other, and the mean value of positive cells/mm^2^ per fetus was calculated and used for statistical analysis. To determine the inter-examiner reliability, 10 sections were measured by the two observers and acceptable coefficient of determination (*R*^2^) scores >0.80 were obtained for cell counting.

### Apoptotic DNA fragments in macrophages

Transversal sections containing palatal shelves in midline adhesion and fusion of wt and HO-2 KO fetuses were selected for the F4/80-immuno staining—FragEL™ DNA fragmentation kit combination. The F4/80 surface receptor is considered as one of the best markers for mature macrophages (Lin et al., 2005). The proximity of macrophages to apoptotic DNA fragments within the MES and the presence of apoptotic DNA fragments within the F4/80 positive macrophages were studied. For used antibodies and antigen retrievals, see Tables 2, 3.

### CXCR3 and HO-1 expression in macrophages studied by immunofluorescence microscopy

Double staining for F4/80 with CXCR3/HO-1 were performed on paraffin sections of wt and HO-2 KO fetuses. Tissue samples were fixed for 24 h in 4% paraformaldehyde and further processed for routine paraffin embedding. Sections were deparaffinized using Histosafe and rehydrated using an alcohol range (100–70%). Fluorescent immunohistochemical double stainings for F4/80 with CXCR3, and F4/80 with HO-1 were performed. Nuclear staining was performed with DAPI. For antibodies used, see Tables 2, 3.

### Statistical analysis

The data for the implantation rate, fetus weight, fetus length, fetus surface, fetus head surface and the PCR data for the mRNA expression of HO-1, HO-2, CXCL11, and CXCR3 showed a normal distribution as evaluated by the Kolmogorov-Smirnov test (KS-test).

To compare differences in implantation rate between the wt group and HO-2 KO group the Independent-Samples *T*-test was performed.

To analyze the fetus weight, fetus length, fetus surface, fetus head surface for the wt E15 group, the HO-2 KO E15 group and the HO-2 KO E16 group the ANOVA and Tukey's multiple comparison *post hoc* test were used.

The HO-1, CXCL11, and CXCR3 immunoreactivity in the epithelium regions was semi-quantitatively scored and analyzed using the non-parametric Kruskal-Wallis ANOVA on ranks test and Dunn's Multiple Comparison *post hoc* test to compare differences between the wt group and HO-2 KO group.

The data from quantification of the number of HO-1 positive cells in the mesenchyme showed a non-normal distribution as measured by the KS-test and the non-parametric Mann-Whitney test was used to compare differences between the wt group and HO-2 KO group.

Quantification data of the number of CXCL11 and CXCR3 positive cells in the mesenchyme showed a normal distribution as measured by the KS-test. Independent-Samples *T*-test was performed to compare differences between the wt group and HO-2 KO group.

To determine the inter- examiner reliability, the coefficient of determination (*R*^2^) was calculated by the square of the Pearson correlation coefficient for the quantitative data, and calculated by the square of the Spearman correlation coefficient for the semi-quantitative data.

Differences were considered to be significant if *p* < 0.05. All statistical analyses were performed using Graphpad Prism 5.03 software (GraphPad Software, San Diego, CA, USA).

## Results

### Fetal growth restriction and malformations occur in the absence of HO-2 expression

**Figure 5:**
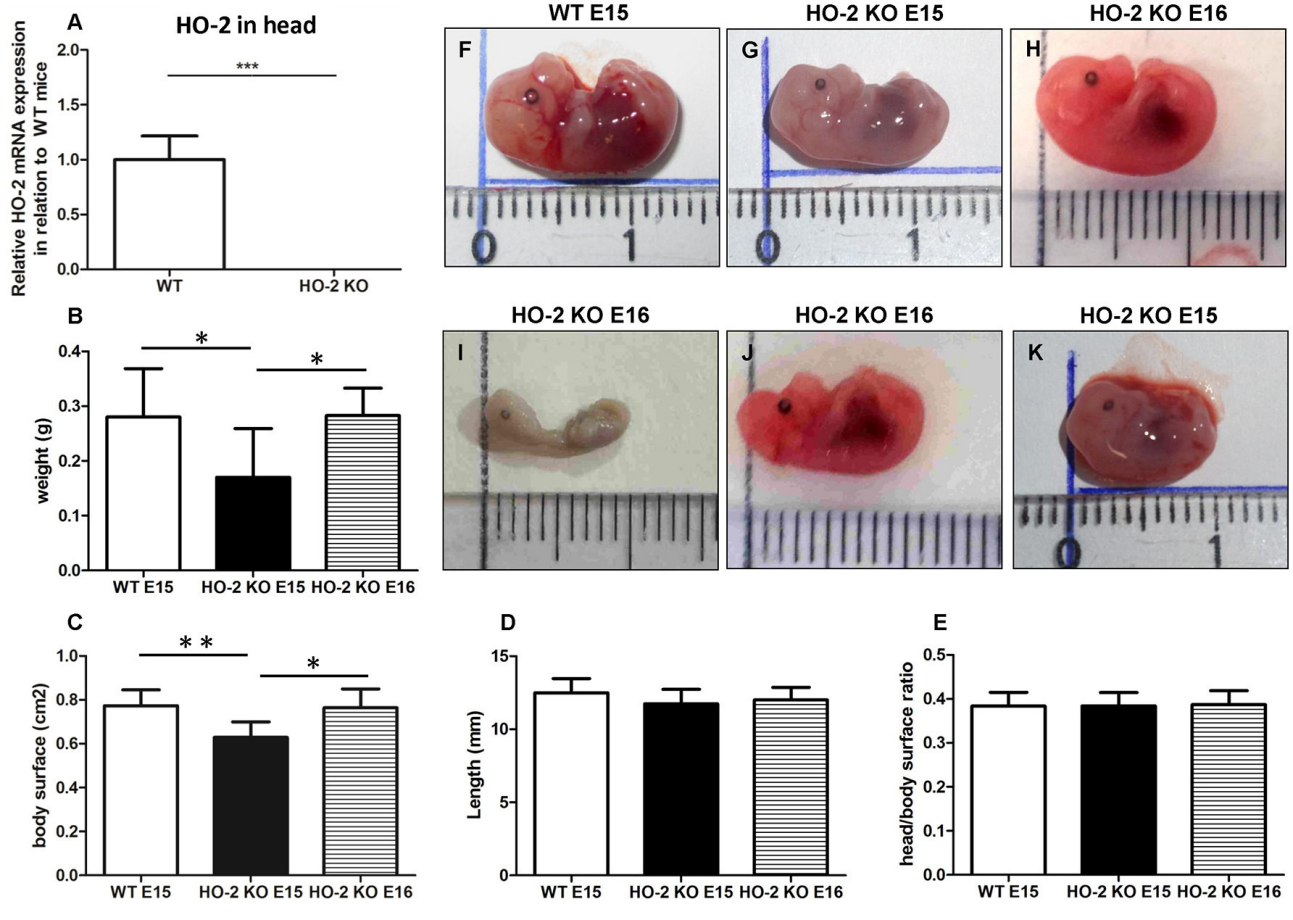
Fetal growth restriction and malformations occur in the absence of HO-2 expression. **(A)** HO-2 mRNA was not found in HO-2 KO fetuses. HO-2 mRNA was observed in wt fetuses E15 (*n* = 5), but not in HO-2 KO fetuses E15 (*n* = 4). Wt fetuses (E15; *n* = 15), HO-2 KO fetuses (E15; *n* = 4), and HO-2 KO fetuses (E16; *n* = 11) were compared for **(B)** weight, **(C)** body surface, and **(D)** length (*P* = 0.25), **(E)** head/body surface ratio (*P* = 0.97). Data presented as mean ± SD. (^*^P < 0.05; ^**^P < 0.01), (^***^P < 0.001). **(F)** wt fetus at E15 (0.28 g; 12.9 mm). **(G)** HO-2 KO fetus at E15 (0.13 g; 12.5 mm). **(H)** HO-2 KO fetus at E16 (0.31 g; 12.4 mm). **(I)** HO-2 KO fetus at E16 demonstrating severe malformations (0.065 g; 10 mm). **(J)** HO-2 KO fetus at E16 demonstrating a craniofacial anomaly (0.20 g, 12.2 mm). **(K)** HO-2 KO fetus at E15 appeared to be the smallest fetus without anomalies (0.10 g, 10.3 mm).

Quantitative real-time PCR confirmed the genotypes of mice by showing that HO-2 mRNA was only present in samples from wild-type (wt) fetuses, and not in HO-2 KO fetuses (*P* < 0.001, Figure 5). Hemorrhagic embryonic implantations were found in both wt and HO-2 KO animals (Figure 1A). No significance difference in the mean implantation rate was found between pregnant wt and HO-2 KO mice (46 vs. 52%, *P* = 0.79). At E15, HO-2 KO fetuses weighed significantly less (*P* < 0.05, Figure 5B) with a significantly smaller body surface than wt fetuses (*P* < 0.01, Figure 5C). The differences fetus length (*P* = 0.25, Figure 5D) and in head/body surface ratio (*P* = 0.97, Figure 5E) for both genotypes were not significantly different at E15. To monitor the restriction of fetal growth in HO-2 KO fetuses in more detail, we also analyzed the body size of E16 HO-2 fetuses. No significant difference was found between the E15 wt fetuses and E16 HO-2 KO fetuses in regards to weight (Figure 5B) and body surface (Figure 5C). No malformations or craniofacial anomalies were found in the wt fetuses (Figure 5F). Among the 15 HO-2 KO fetuses (E15 and E16), 1 E16 fetus had severe malformations (Figure 5I), 1 E16 fetus exhibited a craniofacial anomaly (Figure 5J), and 1 E15 fetus appeared to be the smallest fetus without anomalies (Figure 5K).

### Palatal fusion observed in both wt and HO-2 KO fetuses at E15

Though the HO-2 KO fetuses were smaller in size, no difference in the adhesion and fusion of the palatal shelves was observed between the sections from wt and HO-2 KO fetuses at E15. In 2 wt fetuses and 2 HO-2 KO fetuses at E15, the palatal shelves were not yet elevated. In both genotypes, different phases of palatogenesis were observed in histological sections from the same fetus. The MES changes from a multi-cell layer into a continuous single-cell layer, to a disintegrating MES, during which islands of epithelium are observed. For more details, see Figure 2.

### CXCL11 expression in the MES and mesenchyme

**Figure 6:**
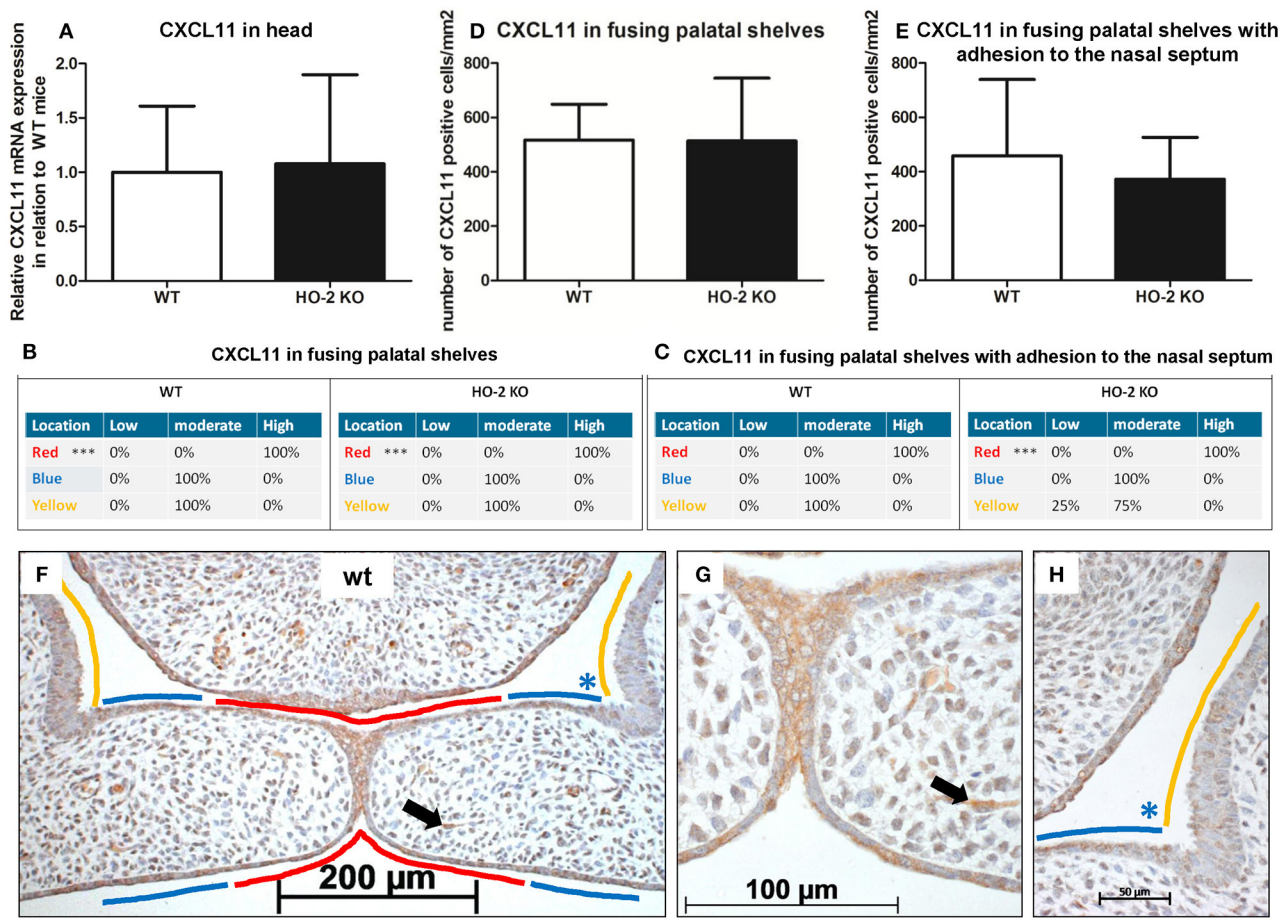
CXCL11 expression in the MES and mesenchyme in both wt and HO-2 KO fetuses. **(A)** CXCL11 mRNA expression was both present in wt (*n* = 5) and in HO-2 KO E15 fetuses (*n* = 4; *P* = 0.88). Data presented as mean ± SD. CXCL11 overexpression in the MES in wt and HO-2 KO fetuses. Scoring was performed according to Figure 3. **(B)** Significant higher CXCL11 expression was observed in the MES (in RED; ^***^P < 0.001) compared to the other epithelial regions in the fusing palatal shelves (in BLUE) and the nasal cavity (in YELLOW) in the wt group and HO-2 KO group. **(C)** Significant higher CXCL11 expression was observed in the MES (in RED; ^***^P < 0.001) compared to the BLUE region and YELLOW region in the HO-2 KO sections with fusing palatal shelves with adhesion to the nasal septum. **(D)** No significant difference in the number of CXCL11 positive cells/mm^2^ in the mesenchyme of the fusing palatal shelves was found between the wt and HO-2 KO fetuses) (*P* = 0.97). Data presented as mean ± SD. **(E)** No significant difference in the number of CXCL11 positive cells/mm^2^ was found in the mesenchyme between the wt and HO-2 KO group in the sections with fusing palatal shelves with adhesion to the nasal septum(*P* = 0.97). Data presented as mean ± SD. **(F)** Representative CXCL11 immunostaining in fusing palatal shelves without adhesion to the nasal septum of a wt fetus (E15) (magnification: x100). The MES (in RED) was highly CXCL11 positive compared to the other epithelial regions; epithelium of the lateral half of the palatal shelves (in BLUE) and epithelium of the lateral wall of the nasal cavity (in YELLOW). **(G)** Several CXCL11 positive cells in the mesenchyme were observed (e.g. black arrow indicates a CXCL11 positive cell in the mesenchyme) (magnification: x400). This was found in both wt and HO-2 KO fetuses. **(H)** Moderate CXCL11 expression in the epithelium of the lateral half of the palatal shelve (in BLUE), and in the epithelium of the lateral wall of the nasal cavity (in YELLOW) (magnification: x400).

Because CXCL11 plays an important role in wound repair, we investigated the mRNA expression of chemokine CXCL11 in fetal head samples. CXCL11 mRNA was observed in samples from both wt and HO-2 KO fetuses without reaching a significant difference between the two genotypes (*P* = 0.88, Figure 6).

Next, we investigated the cellular expression of CXCL11 during palatal fusion. CXCL11 protein was significantly higher expressed in the epithelium of the MES compared to the other epithelial layers of the fusing palatal shelves and the epithelium of the nasal cavity in both wt (*P* < 0.001) and HO-2 KO fetuses (*P* < 0.001; Figures 6B,C,F–H). No significant difference was found between the genotypes. High CXCL11 protein expression was also found within the epithelium of the tips of the palatal shelves lacking midline adhesion, in sections of both genotypes (data not shown). CXCL11-positive cells were also observed in the mesenchyme of the palatal shelves, but no significant difference in the number of CXCL11-positive cells/mm^2^ was found between the wt and HO-2 KO groups (*P* = 0.97, Figure 6D) or in the samples with fusing palatal shelves with adhesion to the nasal septum (*P* = 0.47, Figure 6E).

### CXCR3 expression in the MES and mesenchyme

**Figure 7:**
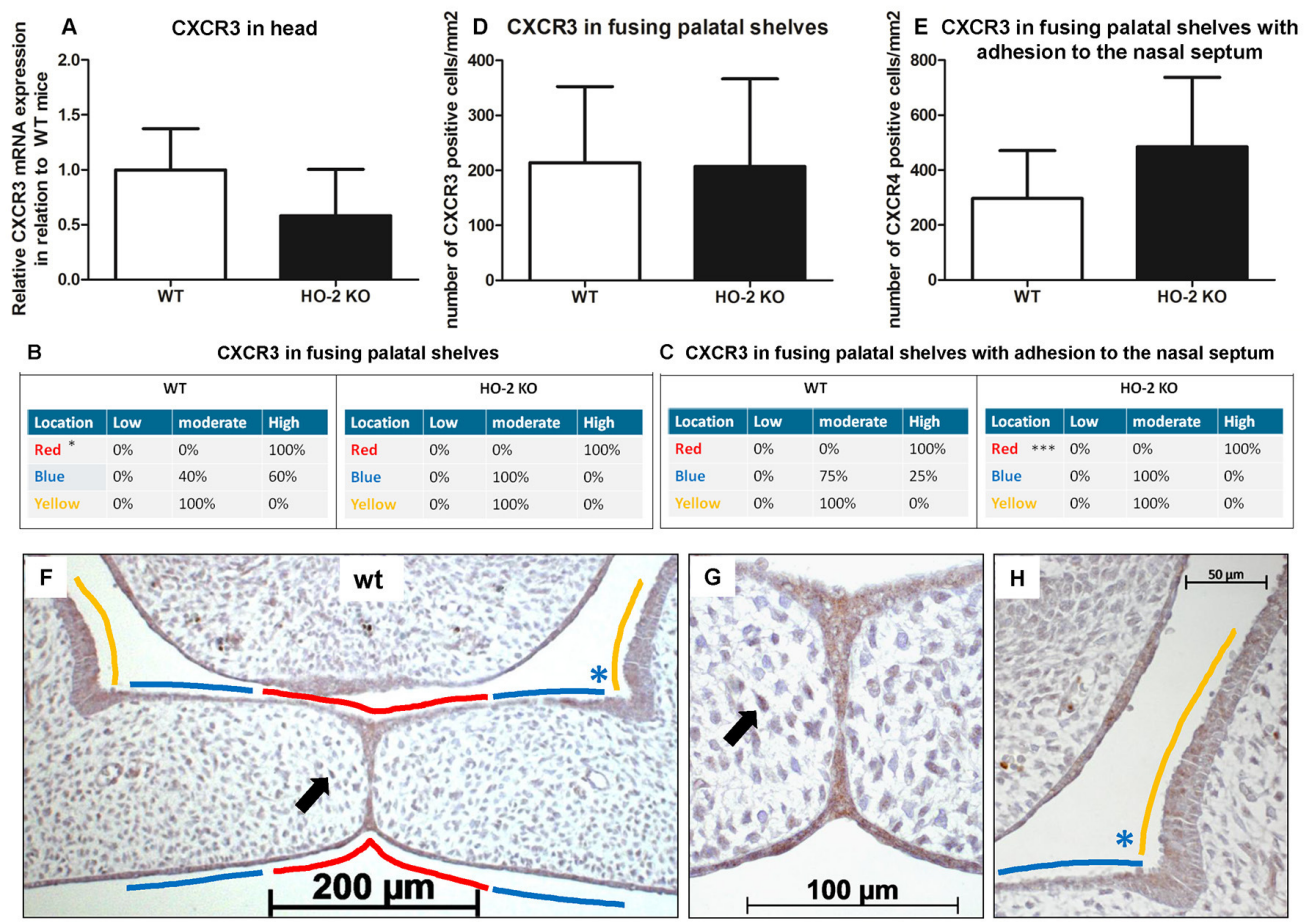
CXCR3 expression in the MES and mesenchyme in both wt and HO-2 KO fetuses. **(A)** CXCR3 mRNA expression was found in wt fetuses E15 (*n* = 5) and in HO-2 KO fetuses E15 (*n* = 4; *P* = 0.16). Data presented as mean ± SD. **(B)** Statistically significant higher CXCR3 expression was observed in the wt group in the MES (in RED; ^*^p < 0.05) compared to the YELLOW region. **(C)** Significant higher CXCR3 expression was observed in the MES (in RED) (^***^P < 0.001) compared to the BLUE region and YELLOW region in the HO-2 KO sections with adhesion of the palatal shelves and adhesion to the nasal septum. **(D)** No significant difference in the number of CXCR3 positive cells/mm^2^ in the mesenchyme of the fusing palatal shelves was found between the wt and HO-2 KO fetuses (*P* = 0.96). Data presented as mean ± SD. **(E)** No significant difference in the number of CXCR3 positive cells/mm^2^ in the mesenchyme was found between the wt and HO-2 KO group of the sections with adhesion of the palatal shelves and adhesion to the nasal septum(*P* = 0.47). Data presented as mean ± SD. **(F)** Representative CXCR3 immunostaining of fusing palatal shelves without adhesion to the nasal septum of a wt fetus (E15) (magnification: x100). The MES (in RED) had higher CXCR3 expression compared to the other epithelial regions (in BLUE) and (in YELLOW). **(G)** Some CXCR3 positive cells in the mesenchyme were observed. This was found for the wt sections and HO-2 KO sections (black arrow indicates a CXCR3 positive cell in the mesenchyme) (magnification: x400). **(H)** Moderate CXCR3 expression in the epithelium of the lateral half of the palatal shelve (in BLUE), and in the epithelium of the lateral wall of the nasal cavity (in YELLOW) (magnification: x400).

Next, we investigated the expression of CXCL11 receptor CXCR3 at the mRNA level. In samples from heads of wt and HO-2 KO fetuses, CXCR3 mRNA expression was observed but with no significant difference between the groups (*P* = 0.16, Figure 7).

CXCR3 protein expression was significantly higher in the epithelium of the MES than the other epithelial layers of the palatal shelves and the epithelium of the nasal cavity in the fusing palatal shelves of the wt fetuses (*P* < 0.05, Figures 7B,F–H). Higher CXCR3 protein expression in the epithelium of the MES was also observed in the fusing palatal shelves with adhesion to the nasal septum from the HO-2 KO fetuses (*P* < 0.001, Figure 7C).

Interestingly, CXCR3-positive cells were also observed in the mesenchyme of the palatal shelves. No significant difference in the number of CXCR3-positive cells/mm^2^ was found between the wt and HO-2 KO groups in the fusing palatal shelves (*P* = 0.96, Figure 7D) or the fusing palatal shelves with adhesion to the nasal septum (*P* = 0.20, Figure 7E).

### CXCR3-positive macrophages were located near the MES and phagocytized apoptotic cell fragments of the MES

**Figure 8:**
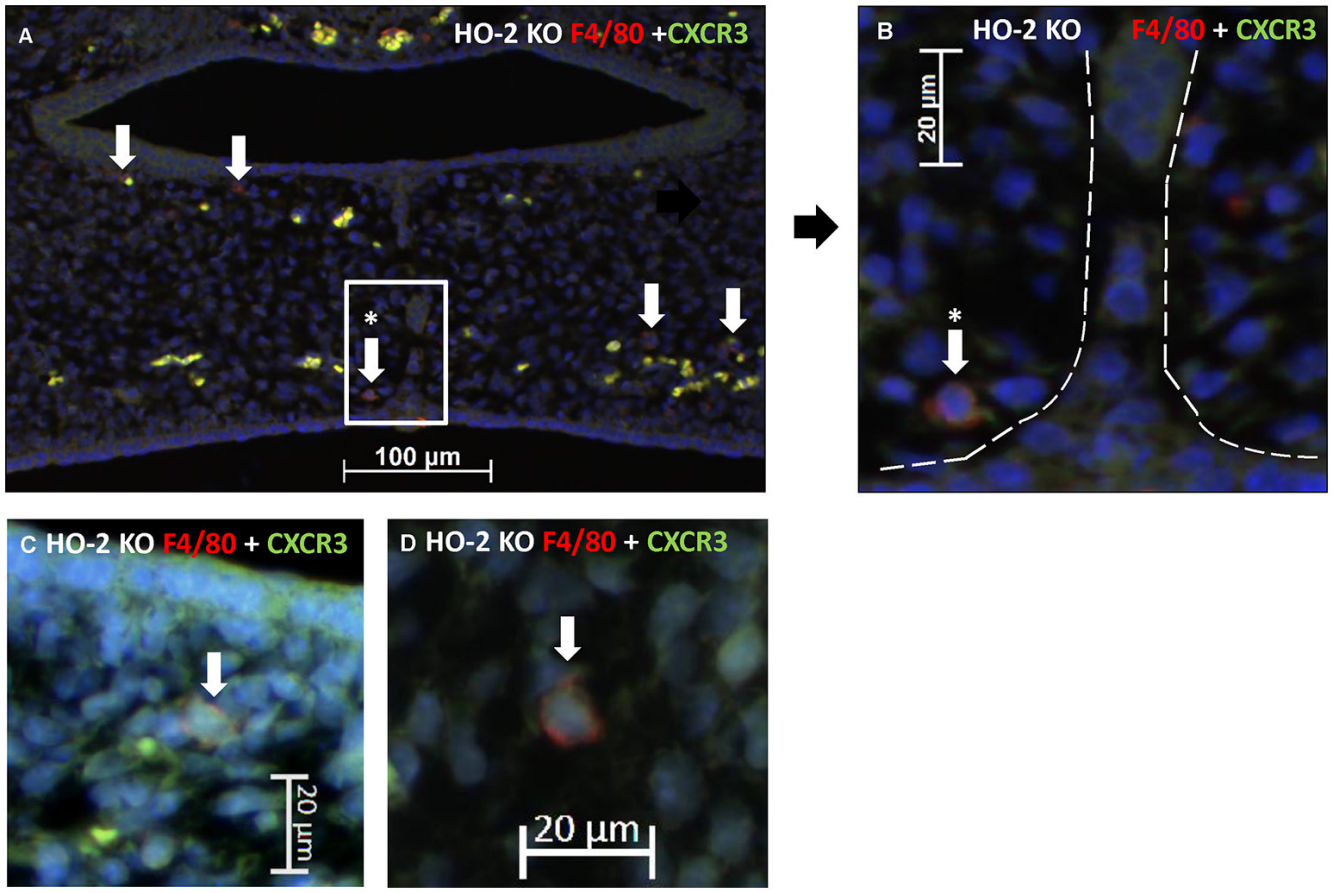
CXCR3 positive macrophages were located near the MES. **(A)** Representative immunofluorescent histochemical double staining for F4/80 (red) with CXCR3 (green) in HO-2 KO section (E16). Nuclear staining with DAPI (Blue). The mesenchyme demonstrates the presence of a F4/80 positive HO-2 KO macrophages, which also express CXCR3 (white arrows) (magnification: x200). One CXCR3 F4/80 positive HO-2 KO macrophage was located near the disintegrating MES (white arrow within the white square). **(B)** Magnification of a F4/80 positive HO-2 KO macrophage located near the MES (area between the dotted white lines) (magnification: x400). **(C)** Magnification of a F4/80 positive HO-2 KO macrophage located in the mesenchym of the palate shelf (magnification: x400). **(D)** Magnification of a F4/80 positive HO-2 KO macrophage located outside the palatal shelf (magnification: x400).

CXCR3-positive cells are suspected of being macrophages based on morphology and because macrophages have been shown to be positive for CXCR3 (Kakuta et al., 2012); (Torraca et al., 2015). Therefore, we investigated whether CXCR3-positive macrophages are present within the fusing palate using immunofluorescence microscopy. F4/80-CXCR3 double-positive macrophages were observed in the fusing palatal shelves, with some located near the disintegrating MES, in both wt and HO-2 KO fetuses (Figure 8).

**Figure 9:**
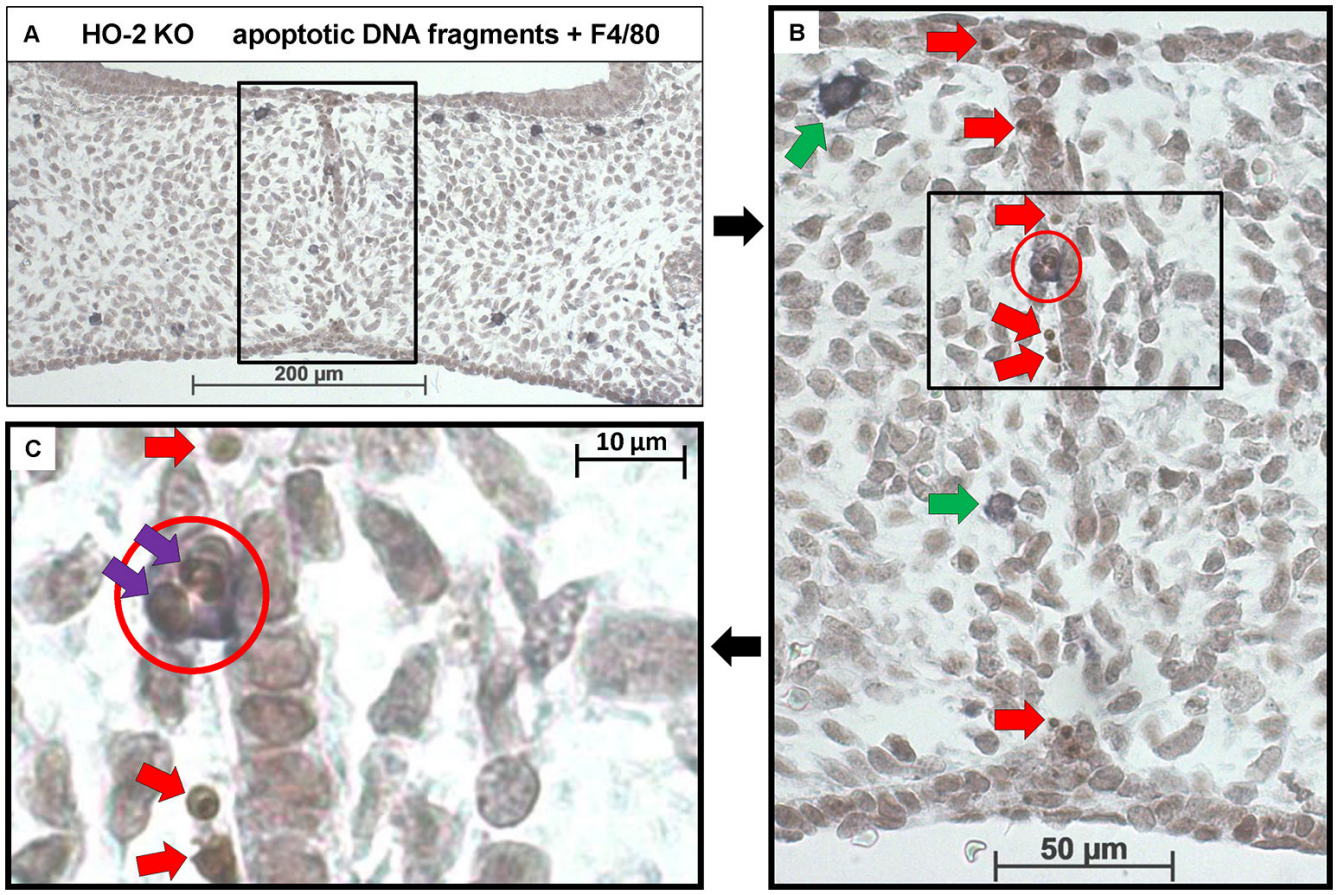
Macrophages located near the MES phagocytose apoptotic cell fragments. **(A)** Representative FragEL™ DNA fragmentation assay (brown) in combination with F4/80 (dark blue) macrophage staining. Fusing palatal shelves in a HO-2 KO fetus (E16) (magnification: x100). Multiple macrophages were observed in the mesenchyme of the palatal shelves. **(B)** Magnification of the disintegrating MES (magnification: x400, black square). Multiple apoptotic DNA fragments are observed within the MES (Red arrows). The only apoptotic DNA fragments in the palatum outside the MES are in macrophages that had taken up epithelial cells seen. Two macrophages were located near the MES (green arrows), one macrophage was in close contact with the MES (red circle). **(C)** Magnification of the macrophage in close contact with the MES (black square). In this macrophage (red circle), two apoptotic cell fragments within its cell body are present (purple arrows). Apoptotic DNA fragments near the macrophage were observed (red arrows). These findings were representative for both wt and HO-2 KO sections.

In both wt and HO-2 KO fetuses, multiple apoptotic DNA fragments were present in the epithelial cells of the disintegrating MES. No apoptotic cell fragments were observed in the other epithelial regions. To assess whether the recruited macrophages phagocytose these apoptotic cell fragments, we stained for both apoptotic fragments and macrophages (FragEL™ DNA fragmentation assay in combination with F4/80). Macrophages located near the MES did phagocytose apoptotic DNA fragments (Figures 9A–C). Other macrophages were observed in the mesenchyme closely localized near the apoptotic DNA fragments within the disintegrating MES (Figure 9B).

### More HO-1-positive cells are found in palatal shelves from HO-2 KO fetuses

**Figure 10:**
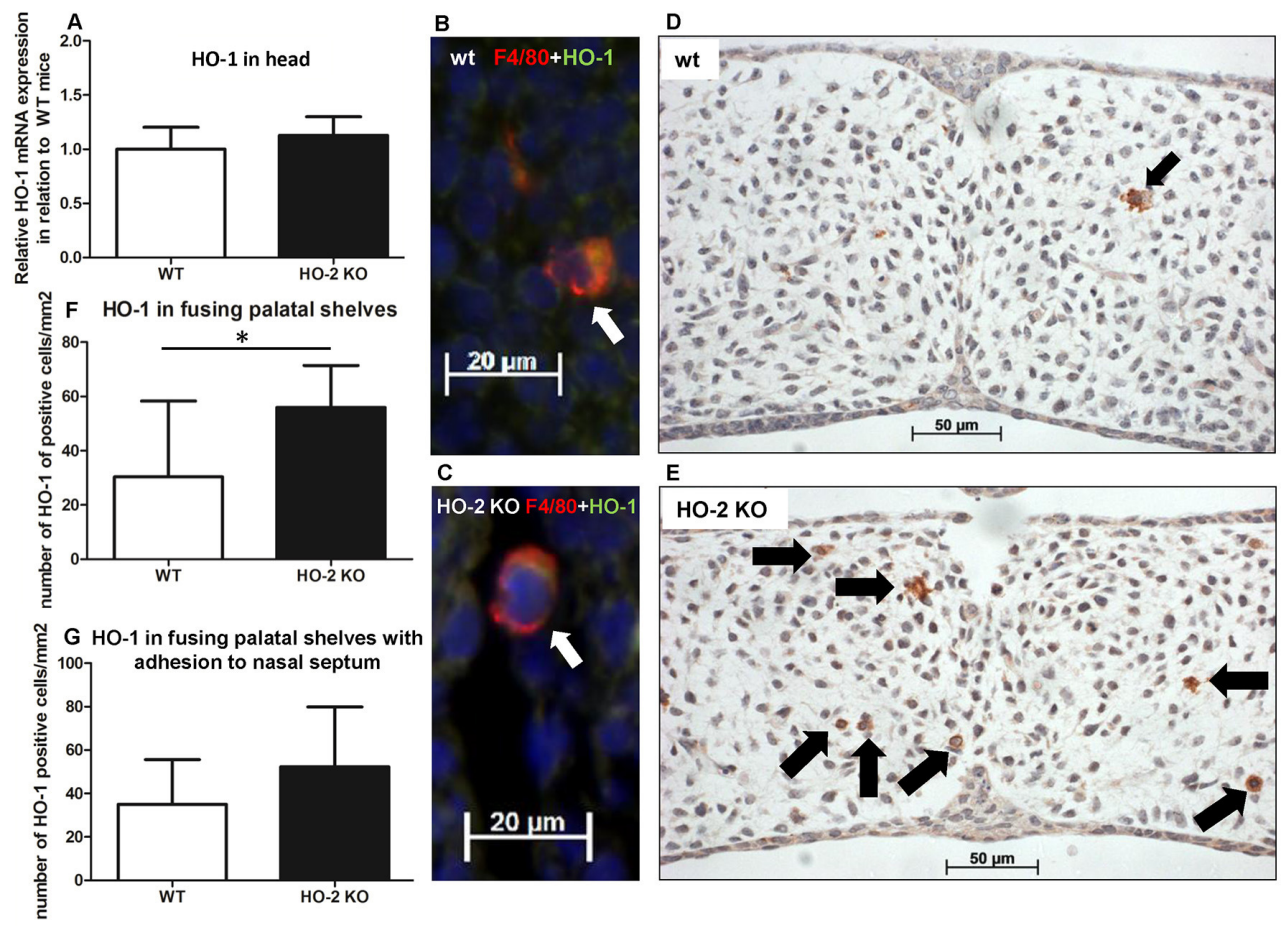
More HO-1-positive cells are found in palatal shelves from HO-2 KO fetuses. **(A)** HO-1 mRNA expression was similar in wt fetuses E15 (*n* = 5) and in HO-2 KO fetuses E15 (*n* = 4; *P* = 0.35). Data presented as mean ± SD. **(B)** Representative fluorescent immunohistochemical double staining for F4/80 and HO-1 of fusing palatal shelves with adhesion to the nasal septum of a wt fetus (E15) (magnification: x400). A part of the mesenchyme around the MES, showing a F4/80 positive macrophage (red) expressing HO-1 (green) located (white arrow). Nuclear staining with DAPI (Blue). **(C)** Representative fluorescent immunohistochemical double staining for F4/80 with HO-1 of fusing palatal shelves with adhesion to the nasal septum of a HO-2 KO fetus (E16) (magnification: x400). A F4/80 positive HO-2 KO macrophage (red) expressing HO-1 (green) located near the MES (white arrow). Nuclear staining with DAPI (Blue). **(D)** Representative HO-1 immunostaining of palatal shelves of a wt fetus (E15) (magnification: x400). This part of the mesenchyme demonstrates the presence of one HO-1 positive cell (black arrow). **(E)** Representative HO-1 immunostaining of fusing palatal shelves of a HO-2 KO fetus (E16) (magnification: x400). This part of the mesenchyme demonstrates the presence of seven HO-1 positive cells (black arrows). **(F)** Significant higher numbers of HO-1 positive cells/mm^2^ were observed in the HO-2 KO fetuses compared to the wt fetuses in the fusing palatal shelves (*P* = 0.02). Data presented as mean ± SD. **(G)** Numbers of HO-1 positive cells/mm^2^ in the mesenchyme of wt and HO-2 KO fetuses in the fusing palatal shelves with adhesion to the nasal septum(*P* = 0.60). Data presented as mean ± SD.

As macrophages can express the cytoprotective enzyme HO-1 during the digestion of cellular debris (Shibahara et al., 1979; Okinaga et al., 1996), we studied whether HO-1-positive macrophages are present within the fusing palate. HO-1 mRNA was observed in samples from the heads of both wt and HO-2 KO fetuses without reaching a significant difference between the two genotypes (*P* = 0.35, Figure 10A). Double immunostaining for macrophage marker F4/80 and HO-1 showed that many F4/80-positive macrophages were positive for HO-1. F4/80 and HO-1-positive cells were observed in the fusing palatal shelves and near the disintegrating MES in both wt and HO-2 KO fetuses (Figures 10B,C).

The number of HO-1-positive cells in the mesenchyme of the fusing palatal shelves was significantly higher in the HO-2 KO group than in the wt group (*P* = 0.02, Figures 10D–G). Almost no HO-1 expression was observed in the epithelium of the palatal shelves in the wt and HO-2 KO groups.

## Discussion

**Figure 11:**
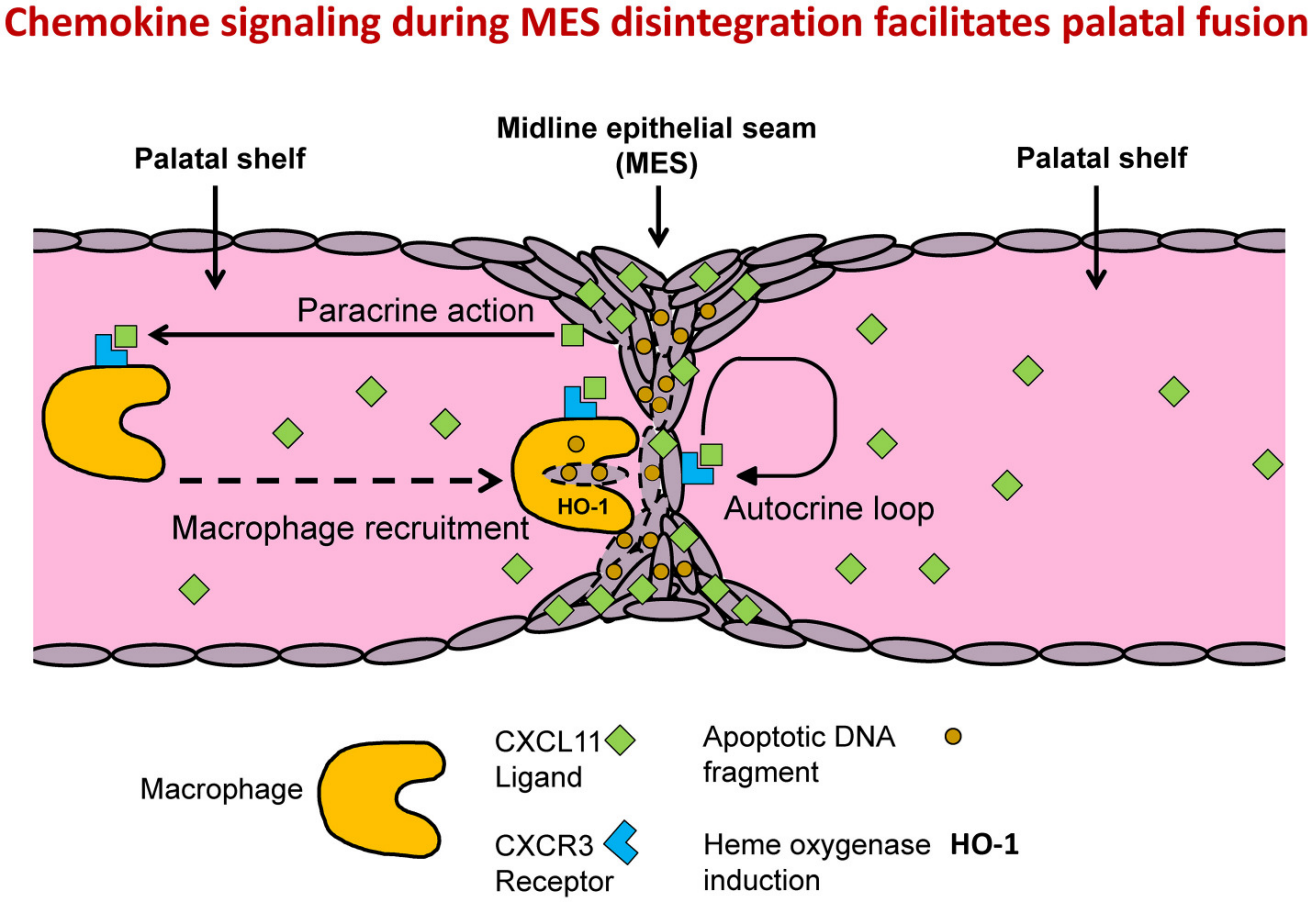
MES mediated chemokine signaling facilitates MES disintegration and palatal fusion. Conceptual model: Autocrine and paracrine MES signaling facilitates palatal fusion. CXCL11/CXCR3 autocrine signaling controls migration and/or apoptosis of epithelial cells during disintegration of the MES. CXCL11-CXCR3 paracrine signaling recruits macrophages to clean up the MESs. HO-2 KO macrophages are still able to phagocytize apoptotic DNA fragments from the MES due to induction of HO-1.

Although deletion of HO-2 expression in mice leads to fetal growth restriction, severe malformations, and craniofacial anomalies, we found no evidence of disruption of palatal fusion in HO-2 KO fetuses. We showed that multiple apoptotic DNA fragments were exclusively present in the MES of both genotypes, supporting earlier findings that apoptosis of epithelial cells drives MES disintegration (Lan et al., 2015). We demonstrated that both CXCR3 and its ligand, the chemokine CXCL11, were highly expressed by epithelial cells in the MES, suggesting that chemokine signaling acts via an autocrine loop to initiate processes involved in its own disintegration. Although, probably also other downstream mechanisms play a role in this process, such as caspases, other enzymes and apoptotic DNA fragments. We demonstrated that apoptotic DNA fragments from the MES were phagocytized by both wt and HO-2 KO macrophages. It is likely that CXCR3-positive macrophages were recruited via CXCL11 expression by the MES (Figure 11). However, we cannot exlude that additionally, other mechanisms can in a later phase facilitate macrophage recruitment. For example, apoptotic nucleotide fragments derived from the MES have been demonstrated to promote macrophage contributed to this recruitment (Elliott et al., 2009). Macrophages near the disintegrating MES were positive for HO-1 in both wt and HO-2 KO fetuses, but more HO-1-positive cells were found in the palate mesenchyme from HO-2 KO fetuses. Although HO-2 KO macrophages have been shown to be dysfunctional in a mouse corneal epithelial debridement model (Bellner et al., 2015), HO-2 KO macrophages phagocytosis of apoptotic DNA fragments still function, possibly with help of HO-1 overexpression.

Adult HO-2-deficient mice are morphologically indistinguishable from wt mice (Poss et al., 1995), but only full-grown mice have been studied thus far. To the best of our knowledge, this is the first study of HO-2 KO embryonic development. Down-regulation of HO-2 is associated with spontaneous abortion in humans (Zenclussen et al., 2003). We did not find that the absence of HO-2 expression resulted in an increased fetal loss rate or decreased implantation rate in mice. However, non-viable and hemorrhagic embryonic implantations were frequently observed in both genotypes. Homozygote HO-2 KO mice were recently demonstrated to be viable (Lundvig et al., 2014), though they demonstrated delayed wound repair (Seta et al., 2006; Lundvig et al., 2014) and an exaggerated inflammatory response after corneal epithelial wounding (Bellner et al., 2011). Fetal growth retardation is associated with down-regulation of HO-2 in human pathologic pregnancies (Zenclussen et al., 2003), which is in line with our findings that HO-2 deletion leads to a developmental growth delay at E15 of approximately 1 day. Among the HO-2 KO fetuses, one was severely malformed and another presented a head anomaly, but no anomalies or malformations were found in wt fetuses.

Environmental factors, such as maternal diabetes, oxidative stress, and infections can have a disturbing influence on palatal fusion and lead to clefting of the lip and palate (Brocardo et al., 2011). HO-2 is essential for regulating physiological levels of reactive oxygen species (ROS) (He et al., 2010; Burgess et al., 2012). Although we found growth restriction and morphological anomalies in HO-2 KO fetuses, proper fusion of the palatal shelves was observed. In the absence of additional stresses, HO-2 KO fetuses can thus develop into mice with a normal palate, possibly due to compensation by elevated HO-1 expression. Next, we studied palatogenesis in HO-2 KO mice in more detail.

We demonstrated increased expression of chemokine CXCL11 and its receptor CXCR3 within the disintegrating MES in both genotypes. CXCR3-CXCL11 signaling serves as a coordinator in wound repair (Huen and Wells, 2012; Kroeze et al., 2012) and is involved in the process of re-epithelialization and epidermis maturation (Lundvig et al., 2014). In keratinocytes, CXCR3 signaling activated μ-calpain to loosen the adhesions for migration (Satish et al., 2005). Scars in CXCR3 KO mice exhibited hyperkeratosis and hypercellularity (Yates et al., 2010), features that are also observed in hypertrophic scar formation in humans (Huen and Wells, 2012). CXCR3 plays a key role in coordinating the switch from regeneration of the epithelial compartment toward maturation (Yates et al., 2008) and modulates cell proliferation and apoptosis (Fulton, 2009; Ma et al., 2015). In the disintegrating MES CXCL11-CXCR3 signaling is therefore likely involved in controlling processes, such as migration and apoptosis of epithelial cells.

We found many apoptotic DNA fragments throughout the disintegrating MES, supporting apoptosis as a driving mechanism in MES disintegration (Vaziri Sani et al., 2005; Xu et al., 2006; Vukojevic et al., 2012; Lan et al., 2015). No apoptotic DNA fragments were found in the other epithelial regions of the palatal shelves or the mesenchyme of the palatal shelves. Blocking cell death with z-VAD, an inhibitor of caspases, leads to persistence of the MES structure, which interferes with fusion of the palatal shelves *in vitro* (Cuervo and Covarrubias, 2004), suggesting that this could lead to cleft palate. However, a role of epithelial migration in MES disintegration cannot be excluded.

In addition to CXCR3 expression in the epithelial MES layer, we also demonstrated CXCR3 expression in the mesenchyme of the fusing palatal shelves. We found CXCR3-positive and phagocytosing macrophages near the disintegrating MES, suggesting that macrophages are actively recruited by CXCL11. This demonstrates that the MES actively participates in its disintegration via chemokine signaling. However, we cannot exclude that other mechanisms play a role, such as caspases, other enzymes and enzyme inhibitors. Recruitment of CXCR3-positive macrophages by CXCL11 paracrine signaling was demonstrated previously also in other models (Kakuta et al., 2012; Torraca et al., 2015).

Although HO-2 deletion impaired macrophage function in corneal epithelial wound repair (Bellner et al., 2015), in our study both wt and HO-2 KO macrophages phagocytosed apoptotic DNA fragments and, thus, were still functional. Although impairment of macrophage function by HO-2 deletion was found in a wound repair study in adult mice (Bellner et al., 2015), we studied macrophage function in a non-pathological environment during embryonic development. However, our findings contradict another wound healing study, demonstrating that HO-2 deletion was associated with impaired HO-1 induction (Seta et al., 2006). Significantly more HO-1-positive cells were found in the palatal mesenchyme of HO-2 KO fetuses compared to wt fetuses, in which HO-1-positive cells were scarce. We suggest that the higher HO-1 expression during embryonic development is a compensating mechanism for HO-2 deletion in recruited macrophages in the fusing palatal shelves. An increased HO-1 induction could explain in part the discrepancy in function between HO-2 KO macrophages in adult and embryonic mice.

A limitation of the present study was the relatively small number of fetuses. However, among the 23 HO-2 KO fetuses, one demonstrated severe malformations and another viable fetus had a craniofacial anomaly, suggesting that HO-2 supports fetal growth and development.

In conclusion, we determined that HO-2 deletion leads to fetal growth restriction and craniofacial anomalies. In contrast to our hypothesis, no disturbance was observed in palatal fusion in HO-2 KO fetuses. However, CXCL11 and CXCR3 were highly expressed in the disintegrating MES in both wt and HO-2 KO animals. Both wt and HO-2 KO CXCR3-positive macrophages were functional since apoptotic cells from the disintegrating MES were phagocytosed. Increased numbers of HO-1-positive cells were found within the mesenchyme of the fusing palatal shelves of the HO-2 KO fetuses. It is tempting to speculate that HO-2 deletion leads to up-regulation of HO-1 expression in macrophages, protecting them from oxidative stress following ingestion of apoptotic epithelial fragments from the disintegrating MES. Our data supports the hypothesis that chemokine signaling by the MES orchestrates its disintegrating by epithelial apoptosis and macrophage recruitment via CXCL11-CXCR3 signaling. However, also alternative pathways may have contributed to these processes. Further research is needed to investigate whether hampered palatal fusion can be the result of disrupted chemokine signaling and whether reduced protection against oxidative and inflammatory stresses promote craniofacial malformations.

## Author contributions

CS: designed experiments, analyzed data, wrote manuscript. NC: performed experiments, wrote manuscript. RvR: performed experiments. RR: provided HO-2 KO mice. PH: performed experiments. SvK: performed experiments. AK: wrote manuscript, supervised research. FW: designed experiments, analyzed data, wrote manuscript, supervised research.

### Conflict of interest statement

The authors declare that the research was conducted in the absence of any commercial or financial relationships that could be construed as a potential conflict of interest. The reviewer JFM and handling Editor declared their shared affiliation.
